# Invasive plant *Alternanthera philoxeroides* suffers more severe herbivory pressure than native competitors in recipient communities

**DOI:** 10.1038/srep36542

**Published:** 2016-11-09

**Authors:** Shufeng Fan, Haihao Yu, Xianru Dong, Ligong Wang, Xiuwen Chen, Dan Yu, Chunhua Liu

**Affiliations:** 1 Department of Ecology, Wuhan University, Wuhan, 430072, P.R. China

## Abstract

Host-enemy interactions are vital mechanisms that explain the success or failure of invasive plants in new ranges. We surveyed the defoliation of invasive *Alternanthera philoxeroides* and co-occurring native plants on two islands during different seasons over three consecutive years and measured the leaf nitrogen content and the C/N ratio of each plant species. To evaluate the effects of herbivory on *A. philoxeroides*, an herbivore exclosure experiment was conducted. We found that the mean defoliation of *A. philoxeroides* was higher than that of native plants, regardless of whether the dominant species was *A. philoxeroides* or native plants. *A. philoxeroides* defoliation increased significantly as the months progressed, whereas the defoliation of the total population of native plants was constant. The leaf nitrogen content was positively correlated with defoliation, and it was highest in *A. philoxeroides*. Additionally, *A. philoxeroides* in the herbivore exclusion treatment showed an increase in shoot biomass and total shoot length. Our study revealed that native generalist herbivores prefer the invasive plant to the natives because of the higher leaf nitrogen content. These results support the biotic resistance hypothesis, suggesting that native herbivore species can limit the population spread of invasive plants.

Novel host-enemy interactions are vital mechanisms that explain the success or failure of exotic plants and mediate the dynamics of invasions in new ranges^1^. Two prominent hypotheses (the enemy release hypothesis and the biotic resistance hypothesis) have been put forward based on these interactions. The enemy release hypothesis proposes that the success of exotic plants is related to their liberation from their specialist herbivores and pathogens and that the enemies in the recipient regions have a greater impact on native plants than exotic plants^2^. Furthermore, when herbivores are absent in the recipient regions, selection will favour genotypes with increased competitive abilities and reduced resource allocation to herbivore defence, so the invasive plants will exhibit faster growth rates and larger sizes (Evolution of Increased Competitive Ability Hypothesis, EICA)^3^. In contrast, the biotic resistance hypothesis proposes that natural enemies in the invaded communities will limit the success of exotic plants because exotic plants usually lack effective defences against native enemies with which they do not share an evolutionary history^4^,^5^,^6^.

Many studies have examined the novel host-enemy interactions in invaded ranges, but the results have been inconsistent^7^,^8^,^9^. Studies have found that exotic plants suffer less herbivory than native plants in the recipient communities and that native generalist consumers prefer native over exotic plants^10^,^11^,^12^,^13^ because the defences employed by the invasive plants are uncommon or absent from the recipient range. Therefore, native generalist herbivores may be deterred or lack a detoxification mechanism^14^,^15^. These studies are consistent with the enemy release hypothesis. However, other studies have shown that native herbivores prefer exotic plants over native plants^6^,^16^,^17^,^18^ because the capability of the exotic plants to express induced resistance to the native herbivores is lower than that of the natives^9^,^19^. The tolerance of exotic plants to damage by native herbivores is also weaker than in natives^9^, so native herbivores can limit exotic plant populations^1^,^20^. These studies support the biotic resistance hypothesis. However, only a few herbivore species have been studied to test the two hypotheses in these study systems, and the preferences of different herbivores for native vs. exotic plants vary, which might have contributed to the inconsistent results. For example, in a study that tested the preferences of aquatic generalist herbivores for exotic or native macrophytes, a native crayfish preferred exotics over natives^18^. In contrast, other studies found that a native snail preferred native over exotic macrophytes, and the larvae of a native moth showed no preference for either native or exotic macrophytes^13^,^21^. Therefore, it is necessary to synthetically evaluate the damage from herbivores to exotic and native plants in recipient communities. Even in field surveys in which the plants suffer attacks from all of the herbivores in the recipient community, a dynamic survey over different seasons is still required because the abundance, richness, and activities of the herbivores vary across seasons^22^.

High leaf nitrogen (N) concentration is thought to confer increased competitiveness because it contributes to a high photosynthetic ability and a relatively high growth rate^23^,^24^. Invasive plants are often characterized as having high leaf N contents and high relative growth rates^25^,^26^,^27^,^28^, but plants with leaves that present high N concentrations are also at risk of preferential consumption by herbivores^29^. Lower nutritive quality has been suggested as an anti-herbivore strategy for plants because low N and water contents have been associated with reduced insect preference and performance^30^,^31^. Studies have found that leaf palatability is positively correlated with leaf N content and water content and negatively correlated with the leaf carbon (C) content and the C/N ratio^27^,^32^. Therefore, invasive plants may be preferentially eaten by native herbivores if they have higher leaf N concentrations compared with the native plants.

*Alternanthera philoxeroides* (Martius) Grisebach (Amaranthaceae) is a perennial herbaceous plant that is both stoloniferous and amphibious. The native range of this species is thought to be the Parana River region of southern Brazil, Paraguay, and Argentina^33^. Currently, *A. philoxeroides* has invaded the USA, China, Australia, New Zealand, Indonesia, India, and Thailand. In invaded areas, *A. philoxeroides* decreases the biomass of native plant species, alters patterns of nutrient cycling, and blocks drainage and irrigation canals, causing economic and ecological problems^31^. In China, *A. philoxeroides* was first introduced into suburban Shanghai from Japan as a forage crop in the late 1930s, and it has now invaded large areas south of the Yellow River Basin and can be found sporadically in northern China. *A. philoxeroides* has been listed as one of the 12 most harmful alien, invasive species in China^34^. In China, more than fifteen generalist insects feed on *A. philoxeroides*^35^,^36^, but there are no reports in the literature discussing the preference of these generalist insects for this invasive species versus native plants or evaluating the effects of native herbivores. In this study, we surveyed the defoliation of *A. philoxeroides* and co-occurring native plants on two islands in different seasons over three consecutive years and measured the leaf N and C contents of the high-frequency plants. Furthermore, an herbivore exclosure experiment was conducted to evaluate the effects of native herbivores on populations of *A. philoxeroides*. We attempted to test three hypotheses: (i) the invasive plant *A. philoxeroides* will suffer more herbivory damage than the co-occurring native plants in invaded communities, and species dominance will affect the preference of the herbivores for *A. philoxeroides* vs. the native plants; (ii) *A. philoxeroides* will present higher leaf N concentrations or a lower leaf C/N ratio than the native plants, which will be a contributing factor to its higher defoliation; and (iii) native herbivores will limit the populations of *A. philoxeroides*. This study contributes to a better understanding of how native herbivores can drive the success of exotic plant species in their new ranges.

## Results

### Does the invasive plant *A. philoxeroides* suffer more damage than the co-occurring native plants in invaded communities?

**Figure 1:**
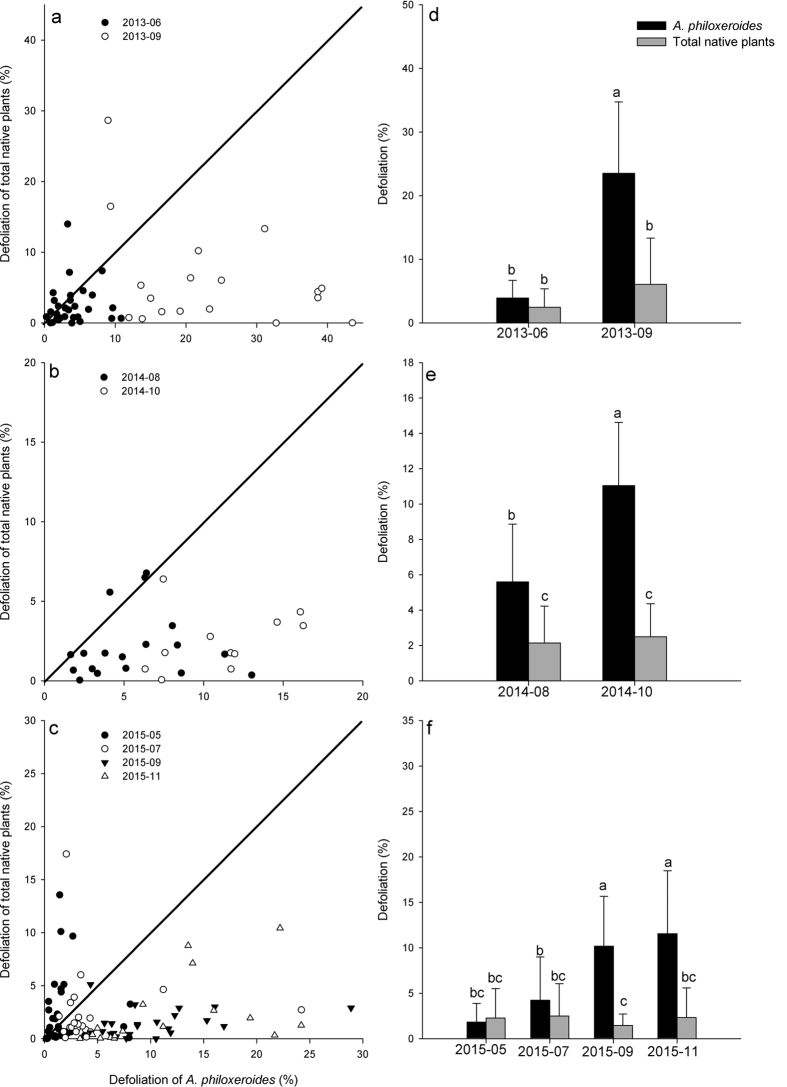
Defoliation of pairs of *A. philoxeroides* vs. the total of the co-occurring native plants in the field surveys in 2013 (**a**), 2014 (**b**), and 2015 (**c**), and the difference in defoliation between *A. philoxeroides* and the total co-occurring native plants in different seasons in 2013 (**d**), 2014 (**e**), and 2015 (**f**). The sloping line in each figure represents the expected 50:50 distribution if the herbivores did not prefer *A. philoxeroides* over native plants.

The results of the three-year field experiments were highly consistent. In the early seasons (June 2013, May 2015, and July 2015), differences in defoliation were not observed between *A. philoxeroides* and the total co-occurring native plants. However, in the later seasons (September 2013, August 2014, October 2014, September 2015, and November 2015), the defoliation of *A. philoxeroides* was significantly higher than that of the native plants, and this difference increased over time (Fig. 1d–f). The significant species × season interactions indicated that differences in the defoliation response to seasons varied among *A. philoxeroides* and the total native plants (Table 1). Over time, the mean defoliation of *A. philoxeroides* increased significantly, whereas the mean defoliation of the total native plants was constant (Fig. 1d–f).

**Figure 2:**
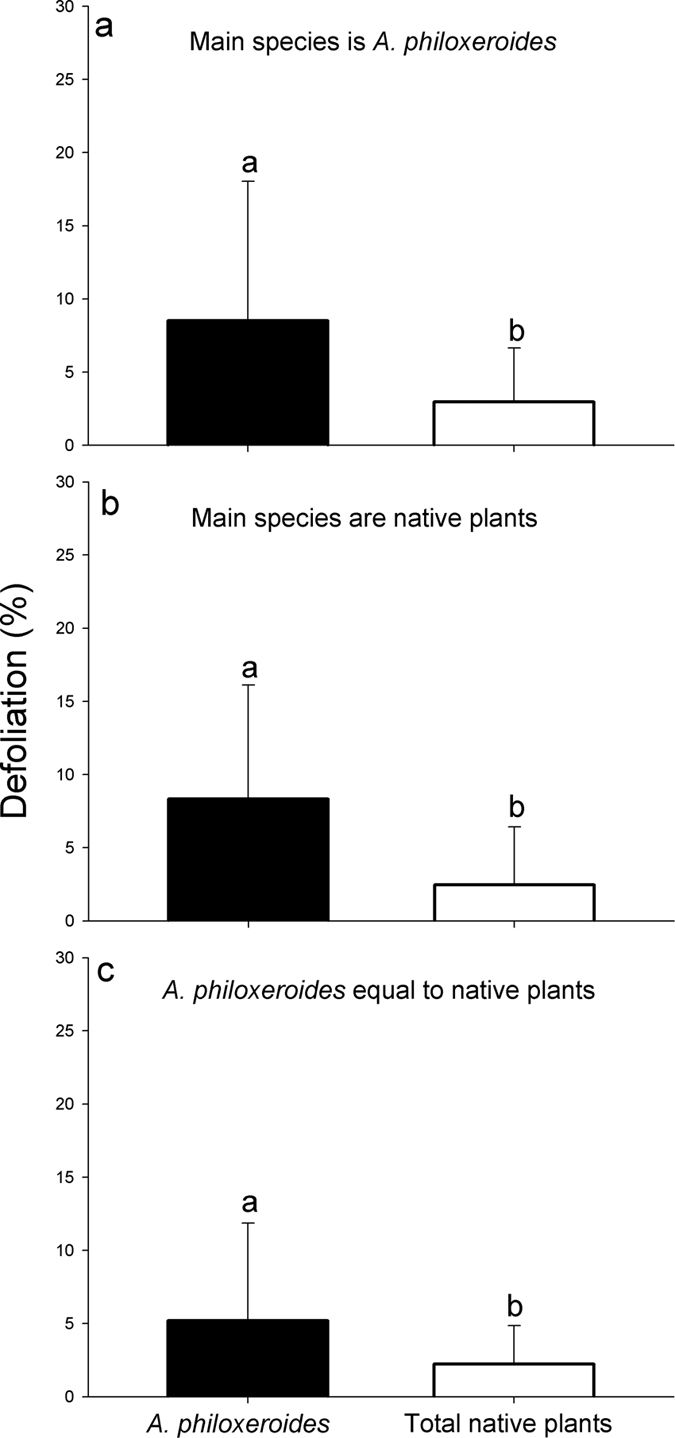
Difference in defoliation between *A. philoxeroides* and the total of the co-occurring native plants in the different communities: (**a**) community in which the main species is *A. philoxeroides* (n = 55), (**b**) community in which the main species are native plants (n = 79), (**c**) community in which *A. philoxeroides* abundance is equal to that of the native plants (n = 40).

**Figure 3:**
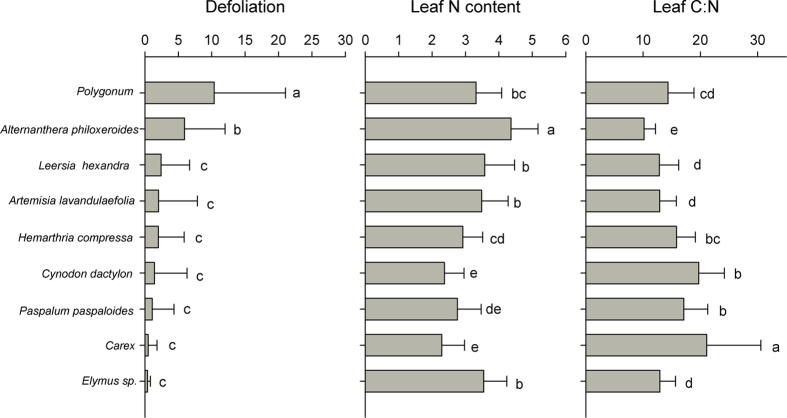
Differences in defoliation (**a**), leaf N content (**b**) and leaf C/N ratio (**c**) in the high-frequency plant species in the 2015 surveys.

In total, 174 quadrats were surveyed over the three study years, and there were 55 quadrats in which the main species was *A. philoxeroides*; 79 quadrats in which the main species were native plants; and 40 quadrats in which the dominance of *A. philoxeroides* was equal to that of the native plants. In all three types of quadrats, the mean defoliation of *A. philoxeroides* was significantly higher than the mean defoliation of the native plants (Fig. 2a, F_1,108_ = 16.216, P < 0.001; Fig. 2b, F_1,156_ = 35.610, P < 0.001; Fig. 2c, F_1,78_ = 6.858, P < 0.05). In the 2015 field survey, the difference in defoliation among plant species was significant (F_8,309_ = 11.040, P < 0.001). The mean defoliation of the native plant *Polygonum* was higher than that of *A. philoxeroides*, whereas the mean defoliation values of the other seven native plants with a high frequency of occurrence (*Leersia hexandra*, *Artemisia lavandulaefolia*, *Hemarthria compressa*, *Cynodon dactylon*, *Paspalum paspaloides*, *Carex*, *Elymus sp*.) were lower than that of *A. philoxeroides* (Fig. 3a).

### Does *A. philoxeroides* present higher leaf N concentrations or a lower leaf C/N ratio, and do these factors contribute to its higher defoliation?

**Figure 4:**
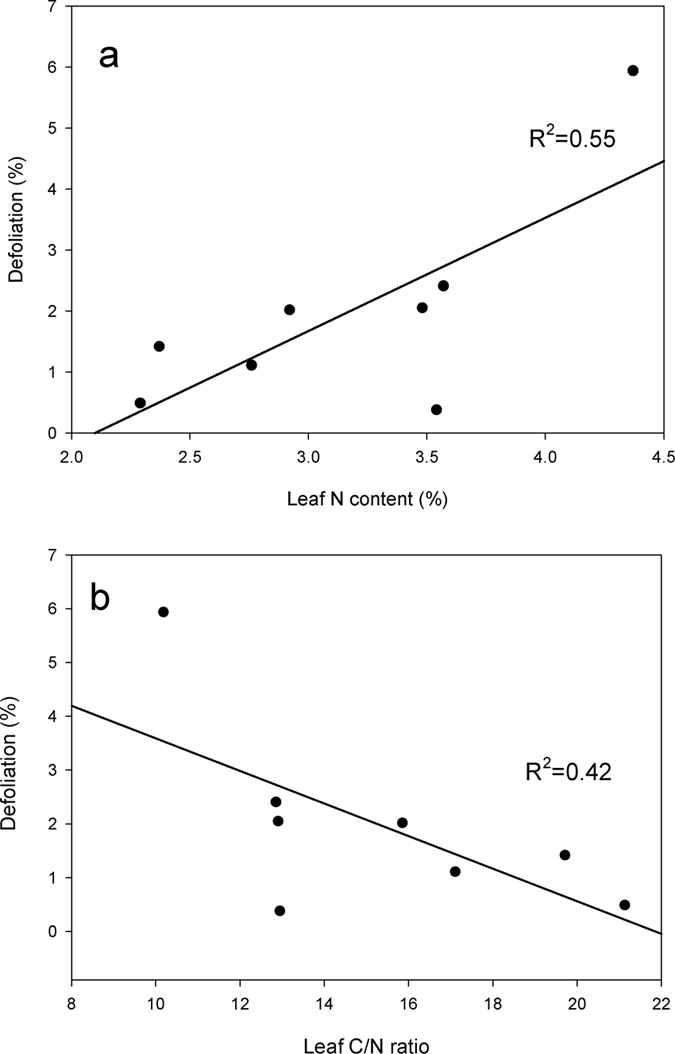
Relationships between mean defoliation and the mean leaf N content (**a**) and mean leaf C/N ratio (**b**).

The difference in the leaf N content among plant species was significant (F_8,199_ = 21.036, P < 0.001), and the leaf N content of *A. philoxeroides* was the highest among all of the evaluated species (Fig. 3b). The leaf C/N ratio also differed significantly among plant species (F_8,199_ = 18.046, P < 0.001); the leaf C/N ratio of *A. philoxeroides* was the lowest of all of the species (Fig. 3c). Mean defoliation value was positively correlated with the mean leaf N content (Fig. 4a, P = 0.035) but negatively correlated with the mean leaf C/N ratio (Fig. 4b, P = 0.08).

### Do native herbivores limit the populations of *A. philoxeroides*?

**Figure 5:**
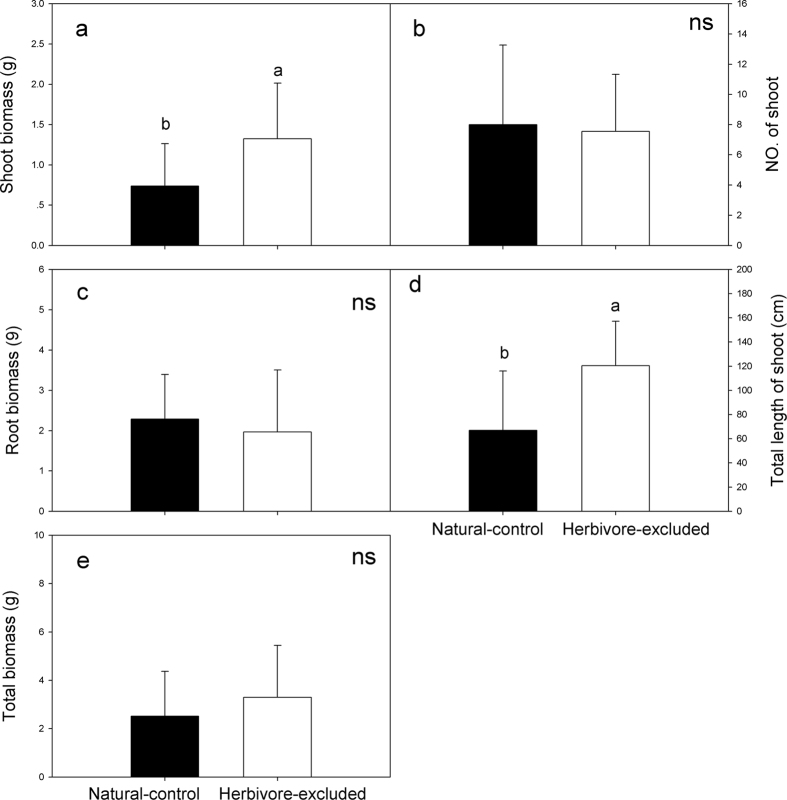
Differences in the shoot mass (**a**), root mass (**b**), total biomass (**c**), shoot number (**e**) and total shoot length (**f**) of *A. philoxeroides* between the natural-control and herbivore-excluded communities; n = 9 for all traits in both treatments, except n = 7 for root mass in the natural-control treatment.

In the natural-control treatment, the specialist beetle, *A. hygrophila*, was absent, so only native herbivores influenced the defoliation and traits of *A. philoxeroides*. The defoliation of *A. philoxeroides* increased over time. In June and July, the defoliation of *A. philoxeroides* was 5% and 2.87%, respectively, although the percentage increased to 12.14% in August. In the herbivore-excluded treatment, all of the leaves of *A. philoxeroides* were intact in the three surveys. After exposure to natural enemies for five months, the shoot biomass of *A. philoxeroides* in the natural-control treatment was significantly lower than that in the herbivore-excluded treatment (Fig. 5a, F_1,16_ = 4.129, P < 0.05), while no differences in the root biomass and the total biomass of *A. philoxeroides* were observed between the two treatments (Fig. 5c, F_1,14_ = 0.21, P > 0.05; Fig. 5e F_1,16_ = 0.667, P > 0.05). Although the number of shoots in the two treatments was similar (Fig. 5b, F_1,16_ = 0.042, P > 0.05), the total length of the shoots in the natural-control treatment was lower than that in the herbivore-excluded treatment (Fig. 5d, F_1,16_ = 6.874, P < 0.05).

## Discussion

Our study supported the biotic resistance hypothesis; the majority of the herbivores in the recipient community preferred *A. philoxeroides* to native plants, regardless of whether the dominant species was *A. philoxeroides* or native plants. In the early seasons, the defoliation of *A. philoxeroides* was not higher than that of the total native plants. *A. philoxeroides* originates from the tropical areas of South America, and Liangzihu Lake is located at the middle and lower reaches of the Yangtze River in the northern temperate zone; thus, the sprouting of *A. philoxeroides* in our field study occurred later than that of the early spring native plants (e.g., *Avena*, *Alopecurus*, *Poa*, *Bromus*, *Beckmannia*, *Carex*, *Elymus*, *Lolium*, etc.). Certain early spring plants had already lost considerable leaf mass through herbivore attacks at the time when *A. philoxeroides* began sprouting. Thus, in certain communities in the early seasons, the defoliation of the total native plants was higher than the defoliation of *A. philoxeroides*. In our study region, two beetles, *Galerucella grisescens* and *Gastroghysa atrocyanea*, appear to be Polygonaceae weed specialists, particularly those of the genera *Rumes* and *Polygonum*^37^. Therefore, the defoliation of *A. philoxeroides* was lower than that of Polygonaceae, but it was greater than that of the other native plants. This suggests that the invasive plants suffered lower herbivory pressure compared with the native plants that were eaten by specialist herbivores. If there are no specialist herbivores feeding on native plants in a recipient community, invasive plants will be subject to higher herbivory pressure than the natives.

In our study, the defoliation of *A. philoxeroides* increased significantly as the months progressed, which might be related to the abundance, richness and composition of the herbivores in the recipient communities because such parameters, especially in the insect community, fluctuated heavily in different months. In two previous studies in China, the abundance and richness of the insect species in temperate grasslands and wetlands increased from spring to autumn; the dominant species and dominancy index value were also different in different months^38^,^39^. The defoliation trend of *A. philoxeroides* was consistent with the dynamics of the insect abundance and richness. Although the abundance and richness of insect species decreased in winter^38^,^39^, the total number *A. philoxeroides* leaves were also reduced in November due to shedding. Therefore, the defoliation of *A. philoxeroides* did not decrease in November.

Primary and secondary plant metabolites have been suggested to reduce the preference or performance of herbivores and determine the palatability of plants^40^,^41^. However, Carmona *et al*.^42^ did not observe an overall association between the concentrations of secondary metabolites and herbivore susceptibility and suggested that genetic variation in the life-history traits of plants (e.g., flowering time, growth rate) was most strongly correlated with susceptibility to herbivores. Leaf N content is positively related to photosynthesis and relative growth rates^23^,^24^, and leaf nutritional qualities may determine plant palatability. Nitrogen is scarce and perhaps a limiting nutrient for many herbivores, so herbivores prefer to feed on plants with higher N contents^29^,^43^. Studies have confirmed that leaf palatability is positively correlated with leaf N content^27^,^32^, and this is consistent with the results of our study, which found that the leaf N content the leaf C/N ratio were positively and negatively correlated, respectively with plant defoliation. To attain a competitive advantage over native plants, successful invasive plants generally have higher leaf N contents than native plants, which improves their resource-use efficiency^26^,^27^,^28^. Consistent with previous studies, our study also found that the leaf N content of *A. philoxeroides* was higher than that of the other native plants, whereas the leaf C/N ratio of *A. philoxeroides* was lower than the native plants. These results suggested that the higher leaf N content contributed to the preference for *A. philoxeroides* by native generalist herbivores.

In the herbivore exclosure experiment, native herbivores were shown to successfully limit the population expansion of *A. philoxeroides* as the exclosure treatment improved the aboveground performance (higher shoot biomass and total shoot length) of the plant. Studies have suggested that native herbivores have a significant and strong negative effect on the establishment and individual performance of invasive species; however, they can only effectively limit the spread of an introduced plant when its density is low^5^,^44^. Herbivory pressure from native generalist herbivores is not sufficient to limit the spread of exotics after they cross a density threshold; therefore, native herbivores fail to completely repel exotic plants^5^,^44^. The evolution of increased competitive ability hypothesis proposes that, in invaded ranges, invasive plants will reduce their protection against specialists but improve their growth and reproduction as well as increase their resistance and tolerance to generalist herbivores^45^,^46^. Therefore, the effects of native generalist herbivores on invasive plants will be reduced. In contrast, Hull-Sanders and colleagues found that invasive plants lose their defence against generalist herbivores in invaded ranges^47^. In our study, the herbivory pressure from native herbivores was considerable, and the defoliation of *A. philoxeroides* increased to 25%. Studies have found that native herbivores can successfully deter the invasion of exotic plants or weak invaders^14^,^17^, although for the pernicious invasive plant *A. philoxeroides*, native generalist herbivores can also reduce the population performance and adverse effects of this species after its successful establishment.

In conclusion, our study found that native herbivores prefer *A. philoxeroides* to native plants because of its higher leaf N content; under pressure from the native herbivores, the aboveground performance and spread of *A. philoxeroides* was inhibited. Our study supported the biotic resistance hypothesis and confirmed that native herbivores could limit the spread of invasive plants. Furthermore, it suggested that native herbivores provide an important ecosystem service by helping to deter the invasion of exotic plants and reduce the adverse effects of invasive species. In addition, because native generalist herbivores prefer invasive plants to natives, the interactions among native herbivores and native plants will be altered, which is a valuable topic for further investigation.

## Methods

### Field surveys

We conducted field surveys on Liangzi Island (114°34′26″–114°35′22″ E, 30°16′13″–30°15′22″N) and Qingshan Island (30°16′44″–30 °15′44″E, 114°33′40″– 114°34′20″N), which are both in the centre of Liangzi Lake (a typical shallow lake in the middle and lower reaches of the Yangtze River); the areas of the two islands are 2.2 km^2^ and 1.0 km^2^, respectively. In the 1980s, *A. philoxeroides* invaded this area, and it is now prevalent in grasslands, gardens, farms, ponds and lake littoral zones on both of the islands. On both islands, with the exception of *Agasicles hygrophila* (the only specialist agent introduced to China for the biocontrol of *A. philoxeroides* from its original range), the native insects *Atractomorpha sinensis*, the larvae of *Cassida nebulosa*, *Hymenia recurvalis*, *Pieris rapae* as well as some aphids and spiders feed on *A. philoxeroides*.

We conducted 8 field surveys over the following months: June and September 2013, August and October 2014, and May, July, September and November 2015. In each survey, communities consisting of *A. philoxeroides* and native plants were randomly selected, but communities in which *A. philoxeroides* was damaged by *A. hygrophila* were excluded. Then, the defoliation of *A. philoxeroides* and the co-occurring native plants was determined in 100 × 100-cm^2^ or 50 × 50-cm^2^ quadrats, depending on the sizes of the communities (100 × 100-cm^2^ quadrats were established in large communities, and 50 × 50-cm^2^ quadrats were placed in small communities). In each quadrat, we checked each of the damaged leaves on one plant and scored them on a nine-degree scale: 1 (full leaf was damaged), 3/4 (most parts of the leaf were damaged), 1/2 (half of one leaf was damaged), 1/4, 1/8, 1/16, 1/32, 1/64, and 1/128. The number of removed leaves (*Nr*) was calculated as the sum of the damaged leaf scores, and we used two methods to assess the defoliation (% leaf area removed) of each plant (*Rd*). If the abundance of the plant was low, the following calculation was used:


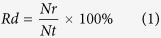


where *Nt* is the total number of leaves on the plant.

If the abundance of the plant was high, the following calculation was used:


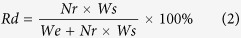


where *Ws* is the mean dry weight of 70–100 random, undamaged intact leaves, and *We* is the dry weight of the extant leaves. The dry leaf weight was determined after drying at 70 °C for 72 h.

The total leaf weight of each plant (*Wt*) was calculated as follows:


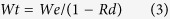


The defoliation of the total of the native plants in the entire quadrat (*Rdn*) was calculated as follows:


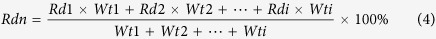


where i is the number of native species in the entire quadrat.

The stem weight of each plant was also determined after drying at 70 °C for 72 h to calculate the shoot biomass of each plant and the total aboveground biomass in the entire quadrat. To identify whether species dominance can affect the preference of herbivores for *A. philoxeroides* and the native plants, we divided all 174 quadrats from the 8 surveys into three types: (1) communities in which the main species was *A. philoxeroides* (the aboveground biomass of *A. philoxeroides* constituted more than 60% of the total aboveground biomass of the entire quadrat); (2) communities in which the main species were native plants (the aboveground biomass of *A. philoxeroides* constituted less than 40% of total aboveground biomass of the entire quadrat); (3) communities in which the abundance of *A. philoxeroides* was equal to the native plants (the aboveground biomass of *A. philoxeroides* constituted 40–60% of the total aboveground biomass of the entire quadrat).

In the four surveys in 2015, 8 native plants (*Polygonum*, *Leersia hexandra, Artemisia lavandulaefolia*, *Hemarthria compressa*, *Cynodon dactylon*, *Paspalum paspaloides*, *Carex sp*., *Elymus sp*.) were high-frequency species (present in more than 16 of the 97 total quadrats). The leaf N and C contents in the undamaged, intact leaves of these plants were measured with a C/N analyzer (FLASH 2000, Thermo, USA). If there were more than 30 samples from one individual plant, 30–40 samples were randomly selected to measure the leaf N and C contents, and if there were less than 30 samples, the leaf N and C contents of all of the samples were measured. At least 16 samples from each plant were measured for a total of 208 samples.

### Herbivore exclosure experiment

To evaluate the effects of herbivory on the populations of *A. philoxeroides*, an herbivore exclosure experiment was conducted on Qingshan Island in 2015. On 8 May 2015, 9 communities without *A. philoxeroides* were selected that were spaced more than 50 m apart in four habitats (two communities under trees, two communities on roadsides, four communities in grasses, and one community in a lake littoral zone). Two 1-m × 1-m plots were established in each community. To maintain the homogeneity of the soil and vegetation, the distances between the two plots in each community were kept within a range of 0.2 m to 1 m. In the centre of each plot, the vegetation was removed from a 0.3-m × 0.3-m area, and six *A. philoxeroides* of similar height and weight (mean height and fresh weight of 40 cm and 3.802 g, respectively) that had been collected from a field population and cultivated for three weeks were planted in each bare area. Each of the plots was randomly assigned to one of two treatments: (1) herbivore-excluded or (2) natural-control. The herbivore-excluded treatment was caged with a screen (1-m × 1-m × 1-m nylon net with 120 mesh and light transmittance of ≥95%), and all of the plants were sprayed with cyfluthrin and DDVP insecticides twice a month to exclude herbivores. The natural-control treatment was in an open natural area where all of the plants were exposed to natural enemies. Two weeks later, we thinned each plot to four similar-sized plants to minimize the plant-size variation among the plots. In the last week of June/July/August, the defoliation of *A. philoxeroides* in both the herbivore-excluded and the natural-control treatments was determined by the previously described method. The experiment lasted five months, and the *A. philoxeroides* was harvested on 8 October 2015. The number and total lengths of the shoots were measured, and the shoot biomass and root biomass were determined after drying at 70 °C for 72 h. The total biomass was the sum of the shoot biomass and the root biomass.

### Statistical analyses

To test our first hypothesis, we evaluated whether *A. philoxeroides* suffered higher levels of defoliation than the co-occurring native plants. In the field surveys, after testing for normality and homoscedasticity, two-way ANOVAS with species (*A. philoxeroides* or native plants), season (different months in one year) and their interaction as fixed factors were employed to analyze the differences in defoliation between *A. philoxeroides* and the total of the co-occurring native plants in the different seasons within each year. Duncan’s tests were then used to compare the levels within factors to determine significance (P < 0.05), and the differences in defoliation between *A. philoxeroides* and the total of the co-occurring native plants in the three types of communities were analyzed using paired t-tests. To evaluate whether exotic plants have higher levels of N or lower leaf C/N ratios, a one-way ANOVA of the differences in the leaf N contents and leaf C/N ratios of these plants was employed, and Duncan’s tests were used to compare the levels within the factors for significance (P < 0.05). To analyze whether the higher leaf N content in plants is a contributing factor to higher defoliation, a linear regression analysis was used to determine the relationships between mean defoliation and mean leaf N content, and mean leaf C/N ratios; however, the native plant *Polygonum* was excluded from these analyses because *Polygonum* plants were eaten by specialist herbivores in the field surveys.

To evaluate the effects of herbivory on the *A. philoxeroides* populations, we used paired t-tests to analyze the differences in shoot number, total shoot length, shoot biomass, root biomass and the total biomass of *A. philoxeroides* between the herbivore-excluded and natural-control treatments in the herbivore exclosure experiment. All of the analyses were performed using SPSS 13.0 (SPSS Inc., Chicago, IL, USA).

## Additional Information

**How to cite this article**: Fan, S. *et al*. Invasive plant *Alternanthera philoxeroides* suffers more severe herbivory pressure than native competitors in recipient communities. *Sci. Rep*. **6**, 36542; doi: 10.1038/srep36542 (2016).

**Publisher’s note**: Springer Nature remains neutral with regard to jurisdictional claims in published maps and institutional affiliations.

## Figures and Tables

**Table 1:** F and P values of defoliation by species (*A. philoxeroides* vs. native plants) and season (two-way ANOVAS).

Source	d.f.	F value	P value
2013			
Species	1.92	52.799	<0.001
Season	1.92	79.559	<0.001
Species × Season	1.92	37.755	<0.001
2014			
Species	1.54	63.280	<0.001
Season	1.54	14.757	<0.001
Species × Season	1.54	11.435	0.001
2015			
Species	1.186	68.285	<0.001
Season	1.186	16.540	<0.001
Species × Season	3.186	18.966	<0.001
